# Cuticular Structures in Micropterous Crickets (Orthoptera, Gryllidae, Petaloptilini, Gryllomorphini)

**DOI:** 10.3390/insects12080708

**Published:** 2021-08-06

**Authors:** Pablo Barranco, José Luis Molina-Pardo

**Affiliations:** 1 Departamento de Biología y Geología, Cite II-B, CECOUAL, Universidad de Almería, Ctra. Sacramento s/n, 04120 Almería, Spain; pbvega@ual.es; 2 CECOUAL, Universidad de Almería, Ctra. Sacramento s/n, 04120 Almería, Spain

**Keywords:** morphology, cuticular structures, troglomorphic adaptations

## Abstract

**Simple Summary:**

Orthoptera, with more than 28,930 described species, is one of the most diverse groups of insects in the animal kingdom. They are divided into two suborders: Caelifera and Ensifera. They have very diverse habits, ranging from epigean to endogean species so that some species exhibit troglomorphic characteristics. A comparative morphological study was carried out by scanning electron microscopy of the different structures of eight species of micropteran crickets whose tegmina had lost their flight and song functionality (Orthoptera, Gryllomorphinae, Petaloptilini, and Gryllomorphini). Special emphasis has been placed on the tegmina and their possible relationship with reproductive functions. In addition, to evaluate troglomorphism in the genus *Petaloptila*, the biometric parameters of six other species have been considered. The existence of structures not previously described in this group (gland openings, setae, pores, or group of campaniform sensilla) was documented, and the relationships between flight loss and stridulation in favor of secretory structures were established. In addition, it has been detected that species of the subgenus *Zapetaloptila* exhibit troglomorphic traits, unlike those of the subgenus *Petaloptila*. This information represents an advance in the knowledge of the morphology of the studied species, especially within tegmina. This also provides information on their degree of troglomorphism and relates to their mode of life. It is a starting point for future research on phylogenetic and histological studies or to find out if the species also inhabit yet undescribed environments.

**Abstract:**

Orthoptera is a very diverse group that has colonized practically all terrestrial ecosystems on the planet. They have adapted to live in the endogenous environment as well as in caves so that some species exhibit troglomorphic characteristics. This group has been extensively studied due to its economic and social importance; however, many basic morphological and biological questions remain to be solved. In this study, a comparative morphological study by scanning electron microscopy of different structures of eight species of micropteran crickets of the tribes Gryllomorphini and Petaloptilini, whose tegmina had lost their flight and song functionality was carried out. Special emphasis was placed on the tegmina and their possible relationship to reproductive functions. In addition, to assess troglomorphism in the genus *Petaloptila*, the biometric parameters of six other species have been considered. Actualization of the lifestyle of the studied species has also been carried out. The results show structures not previously described in this group (gland openings, setae, pores, or group of campaniform sensilla). Structures not previously described in this group have been detected, and tegmina (glandular openings and devoured tegmina) seem to confer a role in reproduction. Troglomorphisms are only observed in species of the subgenus *Zapetaloptila*. Statistically, significant differences have been found in characters such as cephalic elongation, ocular reduction, greater length of appendages, and depigmentation.

## 1. Introduction

Orthoptera is an order of insects that has diversified over some 300 Mya [1,2] and currently has more than 28,972 described species [3]. It comprises two suborders: Ensifera Chopard, 1920, characterized mainly by long antennae, with more than thirty segments, and Caelifera Ander, 1936, with short antennae [1,2,4,5]. Orthoptera has colonized virtually all terrestrial ecosystems on the planet [6], from tropical to arid regions, being absent only in Polar Regions [7,8,9]. They are considered keystone species in food chains because they have a mainly phytophagous diet [4,10] and because they are common prey for other zoological groups, e.g., [11,12,13,14,15]. In some species, population explosions occur that affect crops and can cause enormous economic losses in the agricultural sector [6,16,17,18]. The fact that they are cosmopolitan and this ability to multiply has made this order of insects a traditional food source in different cultures [4,19,20]; thus, they are currently being studied for their nutritional properties [21,22,23]. This group has been extensively studied due to its economic and social importance; however, many basic morphological and biological questions remain to be resolved. In addition, there are a multitude of species in entomological collections worldwide that have not yet been formally described, and a decreasing number of taxonomists are capable of doing the work [6].

Baccetti [24] establishes the group Petaloptilae (syn Petaloptilini) within Gryllomorphini based on the presence of more or less developed tegmina in both sexes, including four genera. Subsequently, after descriptions of new genera [25], synonymies [26] and relocation of some genera [27], the tribe Petaloptilini is currently made up of six genera: *Petaloptila* Pantel, 1890; *Acroneuroptila* Bacceti, 1959; *Glandulosa* Harz, 1979; *Ovaliptila* Gorochov, 2006, and *Hymenoptila* Chopard, 1943 is a subgenus of *Gryllomorpha* Fieber, 1853 (tribe Gryllomorphini) [28]. The genus *Petaloptila* has a mostly Iberian distribution with 12 species, although *P. aliena* (Brunner von Wattenwyl, 1882) is widespread in the French Pyrenees and a single species (*P. andreinii* Capra, 1937) is present in Italy and Corsica. It is subdivided into three subgenera, *Italoptila* Gorochov and Llorente, 2001 for the Italo-Corsican epigean and occasionally hypogean species, *Petaloptila* Pantel, 1890 for the epigean species, and *Zapetaloptila* Gorochov and Llorente, 2001 for the Iberian cavernicolous and endogean species.

Previous research on the taxonomy of the Iberian species of the genus *Petaloptila* [29,30,31] demonstrated that tegmina devoured to a greater or lesser degree (unpublished data). Wing size variation is common in Orthoptera, and almost all subfamilies contain flightless taxa: micropterans or wingless [1,32]. The function of tegmina in the latter cases may be related to other ecological issues [33,34,35]. One of the most studied characteristics associated with Orthoptera is the production of sound, although there are species that have lost this ability. It is used to attract mates, for courtship, or in interactions between males [36] and has caused them to be considered sound bioindicators of ecosystem quality [37]. They produce sound mainly through wing stridulation [6] and tegmino-femoral [5,38], although there are other mechanisms [39,40,41,42,43]. In addition, there are mechanoreceptors in Orthoptera, such as the subgenual organ, which play an important role in the perception of vibrations transmitted by the substrate [44] or the group of campaniform sensilla, which are mechanoreceptors that detect the strain of the cuticle [45,46]. The latter is located close to either the subgenual organ or the tibial organ complex [46]. Copulation in Orthoptera involves the delivery of a sperm sac (spermatophore), which may be accompanied by a nutritional package, the spermatophylax. Males of some Orthoptera also allow females to feed on their body parts during copulation [6].

Orthoptera have adapted to living in the subterranean environment. The species that have colonized this environment, all of which are exclusive to the suborder Ensifera, are characterized by a high degree of endemicity and a higher frequency of relict taxa, although this occurs to a lesser extent than in other arthropods [47]. Most hypogean species are included in the family Rhaphidophoridae, but those belonging to the families Gryllidae, Phalangopsidae, and Trigonidiidae are also prominent [47]. Sometimes, crickets in caves may represent a high percentage of the captured entomofauna, even by being the primary consumers in subterranean habitats [47,48,49,50,51]. Cave fauna is usually classified into three broad categories according to the degree of association with the subterranean environment [52]: trogloxenes, troglophiles, and troglobites. Species that use caves accidentally and, when they do, remain near the entrance are considered trogloxenes; these do not show specific adaptations to this environment [53,54]. Other species, troglophiles, can be found both inside and outside caves and often show adaptations to hypogean life [53,54]. Within this group, Ruffo [55] proposed a subdivision into two categories, later accepted by other authors [53,54]: the eutroglophiles species, being mainly epigean with populations living permanently inside caves and the subtroglophiles species, found in caves but intimately linked to epigean environments in order to perform biological functions (e.g., foraging). Finally, the troglobites species perform their entire life cycle exclusively in subterranean environments. These species are highly specialized in subterranean habitats. Adaptation to life in caves involves morphological, physiological, ecological, and behavioral changes [54]. These traits are often convergent, i.e., they arise independently in different cave taxa under the same set of selective pressures [54]. Furthermore, as suggested by Desutter-Grandcolas [56,57], there may be an evolutionary reversion, and it should be considered that pre-adaptations to cave life (exaptations [58]) may play an important role in their later adaptation to living in the hypogean environment. To refer to common traits of cave-dwelling species, the term troglomorphism is often used [59,60]. However, sometimes it is impossible to classify species by their degree of troglomorphism or by the place where they were found [54,57], and it is necessary to attempt to resolve this by phylogenetic analysis [57]. Nevertheless, the study of troglomorphic characters is still a widely used tool today [47]. According to Desutter-Grandcolas [61,62], the postulated troglomorphic characteristics for crickets adapted to the hypogean environment are reduction in ocular size, depigmentation and elongation of appendages.

In this study, we considered whether the tegmina of Petaloptilini might have a reproductive function as occurs in other Orthoptera species [4,6]. It is also possible that, due to their mode of life, the degree of troglomorphism is greater in the subgenus *Zapetaloptila* because they are mostly troglophiles species, although they can be found frequently in MSS [63]. The subgenus *Petaloptila* species are mainly epigeal, trogloxenes, and MSS [63,64,65,66]. The objectives of this study were (1) to conduct a comparative and mainly descriptive study of the cuticular microstructures of Orthoptera of the tribes Gryllomorphini and Petaloptilini, with special emphasis on tegmina to know if they have a role in reproduction and (2) to evaluate the differences in the degree of troglomorphism within the genus *Petaloptila*. One of the main results of this study is the discovery of a wide range of cuticular structures, mainly in the tegmina, of the tribes Gryllomorphini and Petaloptilini, which are related to their lifestyle and reproductive biology. In addition, adaptations to the endogenous environment have been detected in species of the subgenus *Zetapetaloptila*.

## 2. Materials and Methods

### 2.1. Insects

For the comparative study of cuticular microstructures by SEM, specimens of eight species belonging to the tribes Gryllomorphini and Petaloptilini were considered (Table 1). In addition, to evaluate adaptation in the genus *Petaloptila*, i.e., the acquisition of troglomorphic characters, the biometric parameters of six other species have been taken into account. All the material studied in this study comes from the orthoptera collection of the Entomology Laboratory of the Department of Biology and Geology of the University of Almeria, both the dry materials and other materials were preserved in 70% alcohol.

### 2.2. SEM (Scanning Electron Microscopy)

One to three male specimens of each species have been dissected to study the tegmina, the mouthparts, head, dorsal glands of the thorax, tibiae, and cerci. These structures have been dehydrated and coated with 20 nm of gold (Bal-Tec SCD-005 Metallizer) for observation by scanning electron microscopy (HITACHI S-3500-N Microscope) at a high vacuum. The photographs obtained by this method are marked in the lower left corner with the initials SE. The partially gnawed tegmina of *P. aliena* males have been observed in the SEM without metallization at variable pressure. Photographs obtained by this method are marked in the lower left corner of the photograph with the acronym ESED. The nomenclature proposed by Desutter-Grandcolas [67] is used to describe some of the structures associated with the cerci and that used by Eibl [45] for the structures of the group of campaniform sensilla.

### 2.3. Lifestyles

A live actualization model for the 14 species considered is given. For this purpose, a literature review has been carried out, and unpublished data is provided.

### 2.4. Troglomorphism

The degree of troglomorphism has been studied for the Iberian species of the genus *Petaloptila*, which has species with different ecological habits, both hypogean and epigean. According to Desutter-Grandcolas [61,62], the postulated troglomorphic characteristics for crickets adapted to the hypogean environment are reduction in ocular size, depigmentation, and elongation of the appendages. For this purpose, the coloration annotations of the specimens collected in the field in previous studies have been compiled and confirmed by analysis of the material preserved in alcohol. Likewise, a morphometric study was undertaken in which the following parameters were measured using an ocular micrometer under a stereoscope (in mm): LP, length of pronotum; PF, length of hind femur; PT, length of hind tibia; HH, height of head; HE, height of eye; WE, width of eye. Species have been analyzed using the relationships between these parameters so that the result does not depend on the size of the specimens (it varies in dry as alcohol preserved orthopteran). In the case of eye size reduction, it has been evaluated by relating morphometric indices HH/HE and HE/WE. Appendage elongation was evaluated using the PF/LP and PT/LP ratios. Cephalic elongation was evaluated using the LP/HH ratio.

### 2.5. Statistical Analysis

A statistical analysis was performed using the SPSS for Windows 10.0 (SPSS Inc., Chicago, IL, USA) to determine if there are significant differences in the degree of troglomorphism between the *Petaloptila* and *Zapetaloptila* subgenera. The analysis was performed independently between sexes as their biometrics differ considerably. For this purpose, we first performed a Kolmogorov-Smirnov test for normality and, given that some of the variables do not comply with the principle of normality (α = 0.05, *p* < 0.05), we opted for a non-parametric Kruskal-Wallis test (α = 0.05).

## 3. Results

### 3.1. Study of Cuticular Structure by SEM

#### 3.1.1. Tegmina

**Figure 1:**
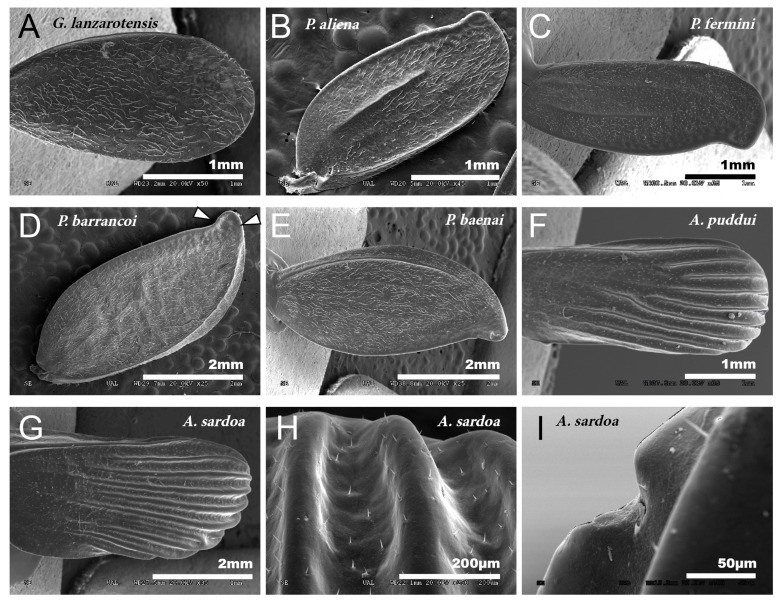
Tegmina. SEM images showing the morphology of the tegmina of male species of the tribes Gryllomorphini, *G. lanzarotensis* (**A**), and Petaloptilini (**B**–**H**). (**A**) Has no notchings or veins. In Petaloptilini (**B**–**H**), both notchings and veins depend on the genus or subgenus. The Iberian species of the genus *Petaloptila* have apical notchings (**B**–**E**) and variable and differently defined veins. *P. aliena* (**B**) and *P. fermini* (**C**) have well-marked veins. In contrast, *Zapetaloptila* has a single vein on the costal margin: *P. barrancoi* (**D**) and *P. baenai* (**E**). (**D**) Two glandular grooves associated with the apical notch of the tegmen are indicated. In the case of the genus *Acroneuroptila* the ribbing forms very pronounced ridges in *A. puddui* (**F**) and *A. sardoa* (**G**,**H**); in addition, there is a very reduced notch (**I**).

The species of the genus *Gryllomorpha* lack tegmina and tympani, except those of the subgenus *Hymenoptila*, which retain the tegmina. The tegmina of the males of *Hymenoptila* are membranous, delicate, without stridulatory apparatus and slight traces of venation [30], covered with a fine pubescence (Figure 1A).

The petal-like flower shape of the male tegmina characterizes the genus *Petaloptila*. These have lost all traces of mirror and stridulatory file, are not membranous but have a thickened appearance. In Iberian species (subgenera *Petaloptila* and *Zapetaloptila*), these tegmina have the apex with a notch in the inner margin (Figure 1B–E). In females of the genus, these structures are reduced to small scales.

Males of the genus *Acroneuroptila* also have highly modified tegmina, without veins or stridulatory files and with a strongly undulated surface due to prominent keels alternating with longitudinal grooves (Figure 1F–H).

In the different species, the development of the veins of the tegmina is very variable. Thus, they are nearly absent in *G. H. lanzarotensis*, and only visible against the light (Figure 1A) but form very prominent ridges in the genus *Acroneuroptila* (Figure 1F–H). Within the genus *Petaloptila*, their development is gradual depending on the subgenus. In the subgenus *Petaloptila*, three veins are seen, two central veins (one internal more marked and the other more external and fainter) and one marginal vein (Figure 1B,C). In the subgenus *Zapetaloptila*, only one vein is visible, coinciding with the costal margin (Figure 1D,E); some longitudinal ones are visible against the light depending on the species. The tegument overlying these veins is similar in design to that seen on the rest of the tegmina surface. Venation in the genus *Petaloptila* is inconspicuous and consists of a slight bulging of the dorsal surface of the tegmina (Figure 1D,E). However, if the tegmina of the species of this genus are viewed in transparency, more longitudinal veins are seen, five–six veins in *P. Z. venosa* [68] and three–five in other species [29]. 

**Figure 2:**
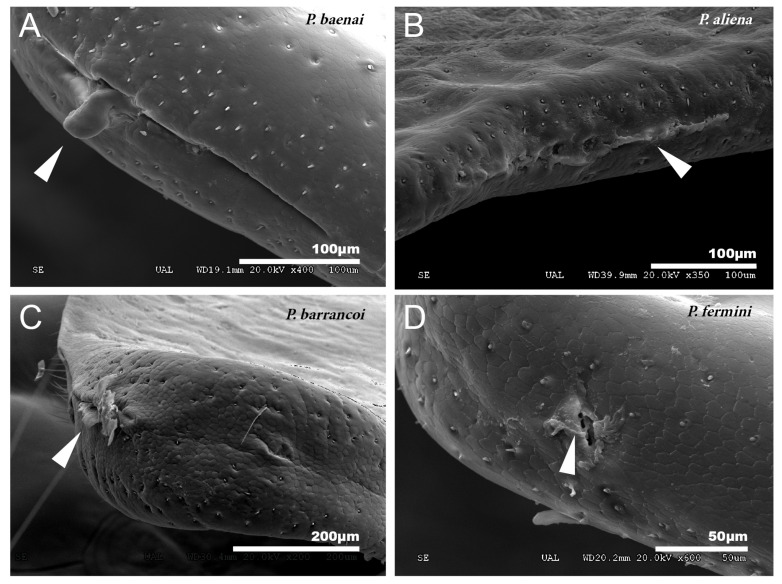
Gland openings. (**A**) Gland openings of *P. baenai*. This is characterized as in the rest of the species of the subgenus *Zapetaloptila* by presenting larger gland openings than in the subgenus *Petaloptila*, *P. aliena* (**B**). The apical gland openings are smaller in *P. barrancoi* (**C**) and *P. fermini* (**D**). The presence of secretion remains is indicated by arrowheads.

In the species of the subgenera *Petaloptila* and *Zapetaloptila,* and the genus *Acroneuroptila* the edge of the tegmina are thick. Gland openings have been detected on the apical edge (indicated by arrowheads in Figure 1D; for more information, Table 2 shows a summary of the main characteristics of each of the eight species analyzed by SEM), in some of which traces of a secretion are observed (indicated by arrowheads in Figure 2A–D). In the genus *Petaloptila,* there are two gland openings, a larger one in the concavity of the notch (Figure 2A,B) and a smaller external one located at the apex of the tegmina (Figure 2C,D). The shape and development of these gland openings differ for each species; thus, the subgenus *Zapetaloptila* have larger gland openings (Figure 2A,B) than those of the subgenus *Petaloptila* (Figure 2B,D).

**Figure 3:**
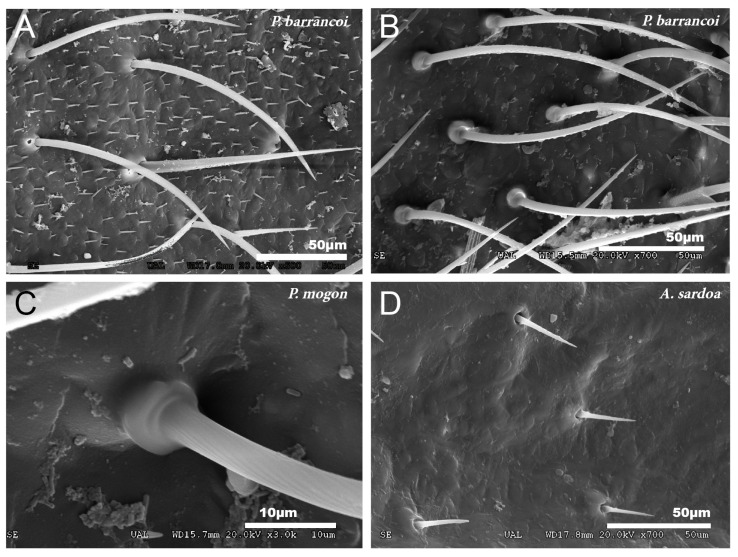
Types of insertion observed in tegmina setae. (**A**) *P. barrancoi*, inserted in a small tumulus; (**B**,**C**) *P. mogon*, inserted in a more developed capsule-shaped tumulus and (**D**) *A. sardoa*, inserted directly on the pore.

No particular structures have been detected on the surface of the tegmina of *G. lanzarotensis,* while the genera *Acroneuroptila* and *Petaloptila* show tegumentary structures and modifications. On the external face of these genera, at 25–45×, a pubescence constituted by microtrichia is observed in most species. This is denser and with longer setae in the species of subgenus *Zapetaloptila* (Figure 1C–E) compared to the species of subgenus *Petaloptila* (Figure 1B), which is shorter, thinner, and sparse. At higher magnification, 500–700×, it can be seen that these thin and acute filiform setae are inserted in a hole on a small mound, in which they can articulate (Figure 3A). This tumulus is more developed and forms a capsule in *P. mogon* (Figure 3B,C). In species of the genus *Acroneuroptila*, the setae are fine and sparse microtrichia and are not inserted on any prominent structure but directly into a pore on the surface (Figure 3D).

**Figure 4:**
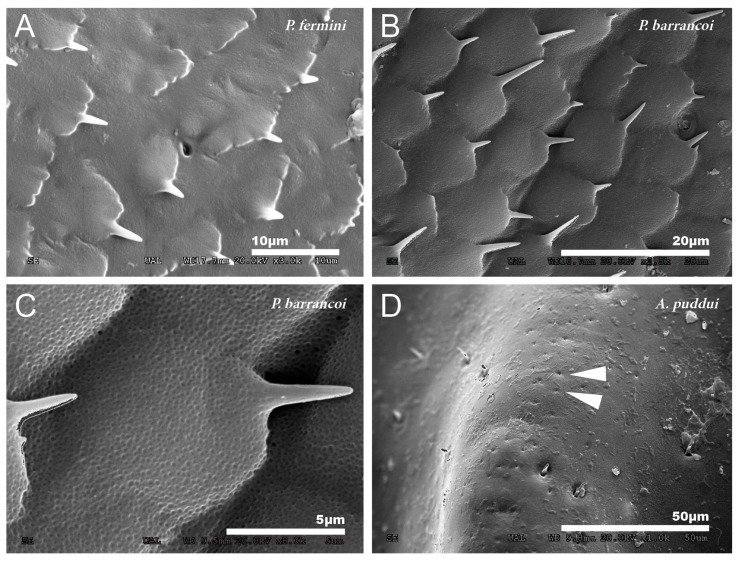
Detailed cuticular study of the dorsal side of the tegmina. Reticulum of imbricate hexagonal scales observed in *P. fermini* (**A**) and *P. barrancoi* (**B**). Most of these scales bear a spine distally, and scattered pores are also present. Dotted surface observed on the surface of the tegmina of *P. barrancoi* (**C**), almost as smooth as in *A. sardoa*. (**D**) Aligned pores observed in *A. puddui*. Arrowheads indicate the presence of pores.

At a magnification of 2500–3000×, the cuticular pattern of the external surface of the tegmen is observed. This consists of a reticulum of imbricate hexagonal scales, the outer margin of which usually bears a spine (Figure 3A and Figure 4A,B), although sometimes there are multiple in number. The outline of this mosaic can be more or less defined (Figure 4A,B). At 8000×, a dotted surface is visible in *P. barrancoi* (Figure 4C). Interspersed among the polyhedral mosaic are widely scattered secretory pores in some of the *Petaloptila* species (Figure 4A). In *G. lanzarotensis* no pores have been located on either the dorsal or ventral side. In the two *Acroneuroptila* species, these pores are very abundant and regularly distributed, both on the dorsal side of the ridges and in the valley between them, forming aligned rows in *A. puddui* (Figure 4D). 

**Figure 5:**
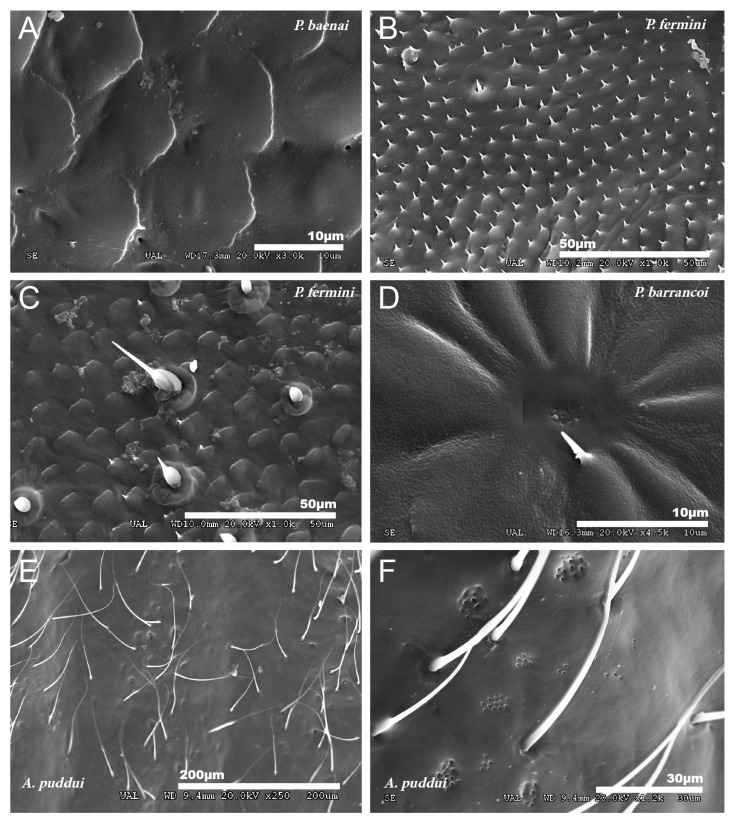
Ventral side of tegmina. (**A**) Less polyhedric design and spiniform than dorsally in *P. baenai* and punctually developed in *P. fermini* (**B**). (**C**) Ampuliform setae in *P. fermini*. (**D**) Pore array in a radial pattern of diffusion grooves in *P. barrancoi*. (**E**) Ventral surface of *A. puddui*; smooth and irregular with finer and longer setae than dorsally. Pores are highly developed in this genus and appear punctually in clusters integrated into concavities like those observed in the SEM image of *A. puddui* (**F**).

The ventral side of the tegmen generally presents a less polyhedral mosaic design, more diffuse, with the margins of the cells more rounded and with less developed spiniform processes (Figure 5A) or punctually developed in some areas (Figure 5B). Although in some species, the tegumentary design is very variable from one part of the ventral side of the tegmen to the other, even the mosaic design disappears. In *G. lanzarotensis,* there is also variation in the mosaic design, from very spiny to very irregular with few spines. In the genus *Petaloptila,* only ventral setae appear in *P. fermini* towards the apex of the tegmen and are ampuliform in appearance (Figure 5C). The presence of secretory pores on this face of the tegmen is general in both *Petaloptila* subgenera, although in some species, more complex structures consisting of a set of pores in the center of a radial design of diffusion grooves have been detected (Figure 5D). In species of the genus *Acroneuroptila,* the ventral surface is smooth and irregular, with some finer and longer setae than on the dorsal side (Figure 5E). The profusion of secretory pores is very pronounced in these species, and they can appear isolated, grouped, and even in numerous groups that can be located in small concavities (Figure 5F).

**Figure 6:**
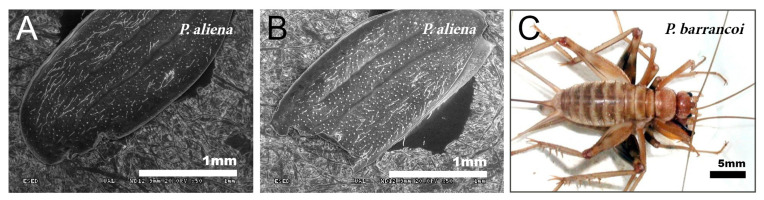
Devoured tegmina and copula in *Petaloptila*. SEM pictures showing incomplete tegmina in *P. aliena*, one more incipient (**A**) and the other with a higher degree (**B**). The image on the left (**C**) shows copulation in *P. barrancoi* in which the female stands on top of the male and the male flips the tegmina forward.

Male specimens with partially devoured tegmina have been found in different species of the genus *Petaloptila*, such as in *P. aliena*, *P. malacitana,* and in another new species under study. The degree of tegmina bite is variable, from incipient (Figure 6A) to consuming half of the tegmina (Figure 6B) and even more.

#### 3.1.2. Legs

**Figure 7:**
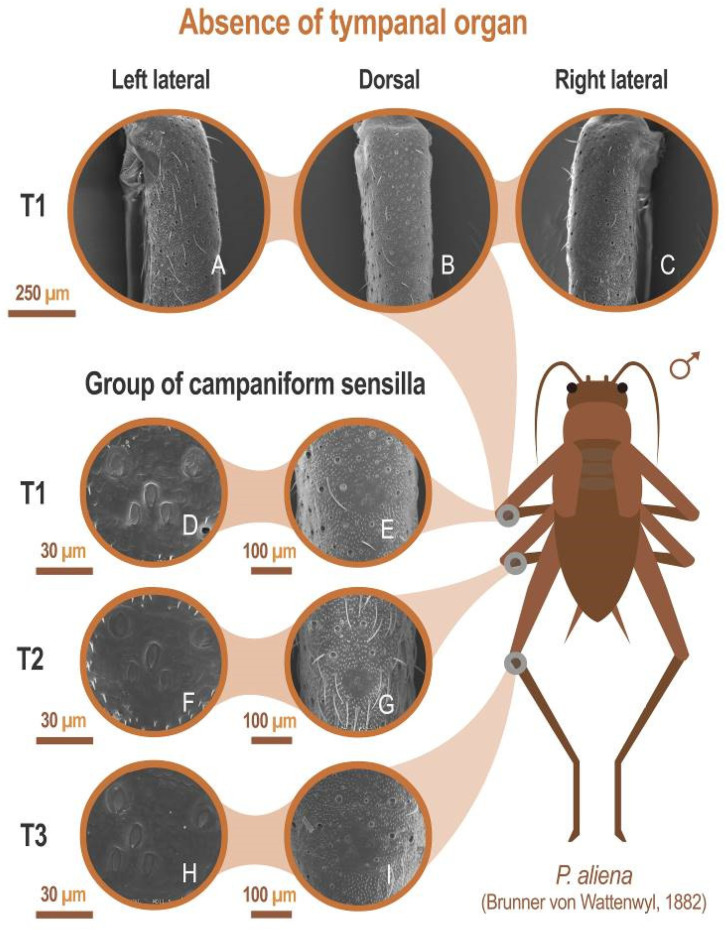
Absence of tympanic organ. SEM images showing the absence of tympani in the anterior tibia (T1) (**A**–**C**), and at different magnifications, the presence of clusters of flared sensilla in the proximal third of the tibiae anterior (T), middle (T2), and posterior (T3) tibiae (**D**–**I**). All SEM images have been performed on the left tibiae from the same specimen. Laterals (**B**,**C**) have been made with a 40° inclination. Specimen preserved in alcohol at 70% of the Orthoptera collection of the Entomology Laboratory of the Biology and Geology Department of the University of Almería. *P. aliena* Serrella (Sierra Serrella, Alicante, Spain), 10/2012. In all cases: P. Barranco col. and det.

All species included in this study lack tympani in fore tibiae (absence of tympanic membranes or their remnants, see, for example, *P. aliena*: Figure 7A–C). All were found to possess a group of camp troglophiles aniform sensilla on all three pairs of legs (see for example *P. aliena*: Figure 7D–I).

**Figure 8:**
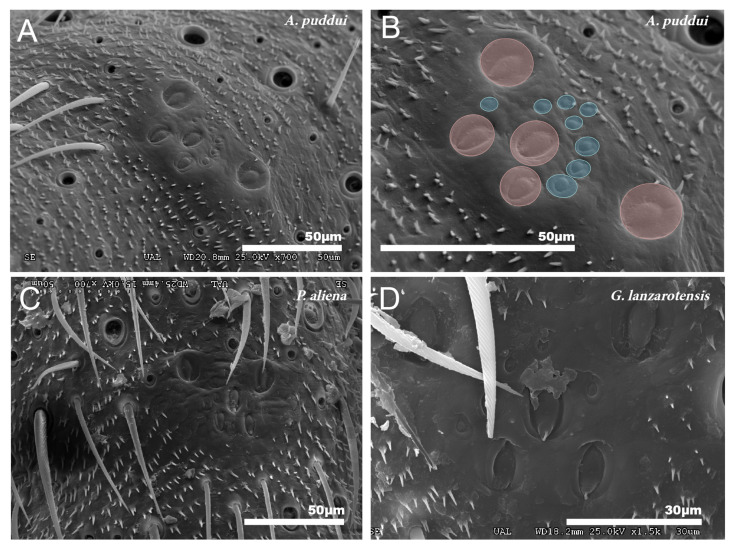
Group of campaniform sensilla. (**A**,**B**) Group of campaniform sensilla detected in the anterior tibiae of *A. puddui*. (**C**) Group of campaniform sensilla of *P. aliena*. (**D**) Group of campaniform sensilla of *G. lanzarotensis*. These types of structures have been detected in all eight species studied by SEM. The design is similar in all cases. In the detailed image of *A. puddui* (**B**), different regions were colored to better highlight the different areas. In pink is the area occupied by the five bell-shaped sensilla arranged in “W”. Just next to these five elements, there is a second area (colored in blue) that is occupied by eight smaller papillae.

These organs consist of a set of campaniform sensilla located in the first third of the tibiae of the three pairs of legs. All cricket species studied consist of five larger sensilla that together form a “W” design. In addition to these sensilla, there are 7–11 small sensilla (Figure 8A–D).

#### 3.1.3. Dorsal Glands

**Figure 9:**
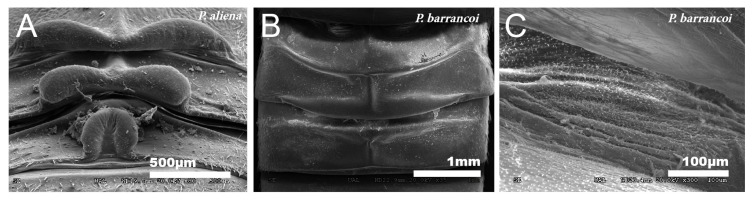
Dorsal glands. SEM pictures showing the dorsal glands in males of the genus *Petaloptila* (**A**–**C**). Within the subgenera of the taxon, they appear more developed and with variable structures and characteristics of each species in the subgenus *Petaloptila*; in the image, we observe those present in *P. aliena* (**A**). In the subgenus *Zapetaloptila,* there is a reduction in the structures, as can be seen in *P. barrancoi* (**B**). The secretory surface is similar in all cases studied. (**C**) SEM image of the secretory surface in the interguite membrane, *P. barrancoi*.

All males of the *Petaloptila* species have dorsal glands in the meso- and metanotum [Table 2], whose conformation has taxonomic value [68,69]. These are much more developed and present structures that are more variable in subgenus *Petaloptila* (Figure 9A) than in subgenus *Zapetaloptila* (Figure 9B). Externally, a reduction or simplification of structures from subgenus *Petaloptila* to *Zapetaloptila* can be considered to have occurred. In the species of the subgenus *Petaloptila,* there is great development and dilatation of the posterior margins of these tergites that form folds with characteristic designs for each species (Figure 9A) [68]. In the hypogean species of the subgenus *Zapetaloptila*, there is a reduction in the size of the folds, which are simpler (Figure 9B) [68]. In both subgenera, the secretory surface of these glangulae consists of a set of grooved folds located on the intertergite membrane of these three segments and is not visible dorsally (Figure 9C).

#### 3.1.4. Cerci

**Figure 10:**
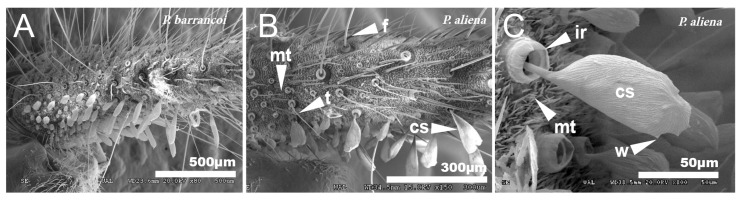
Cerci. SEM pictures showing the surface of the cerci of *P. barrancoi* (**A**) and *P. aliena* (**B**,**C**). The surface is covered with setae of different typologies: cs, globose setae; ir, internal ridge of globose setae; f, filiform setae; mt, microtrichia, t, tricoidsetae; w, crest.

The cerci of this group of species are very long and profusely covered with setae of different typologies [67]. In general, the entire surface is covered with short setae or microtrichia (mt), between which appear long setae called filiform (f) that are inserted in pores with a rim, dotted with very thin tricoid setae (t). In the proximal half of the inner side of the cerci, there are globose setae (cs) that are smaller in the basal part and increase in size towards the end (Figure 10A,B). When comparing the cerci of *P. aliena* with those of *P. barrancoi*, there are differences in the shape of the larger setae. Thus, the distal cs of *P. barrancoi* are longer and more stylized than *P. aliena*, which are even bilobulated. The surface of these setae is striated and has small ridges or crests (w) (Figure 10C) or spines. The insertion of the setae cs has an internal ridge (ir). In general, there are more filiform setae (f) and of greater length in *P. barrancoi* than in *P. aliena*.

### 3.2. Lifestyles

A live update model is provided for the 14 species considered based on bibliographic and own data. Advances in the study of the MSS are providing new and valuable information on the occurrence of epigean species such as troglobias. The results obtained are shown in Table 3.

### 3.3. Troglomorphic Adaptations in the Genus Petaloptila

Troglomorphisms are only seen in species of the subgenus *Zapetaloptila*. It occurs in aspects such as cephalic elongation and ocular reduction, depigmentation, and longer appendages.

#### 3.3.1. Cephalic Lengthening and Ocular Reduction

**Figure 11:**
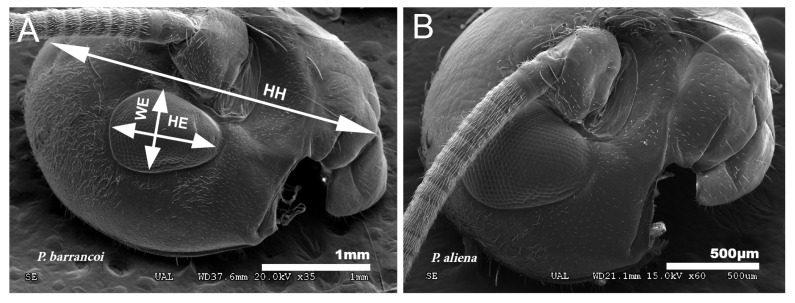
Ocular reduction. (**A**) Reduced eyes in *P. barrancoi* in relation to those of *P. aliena* (**B**). To evaluate ocular reduction, the variables indicated in the image (**A**) were related: HH, head height; WE, eye width; HE, eye height.

The analysis of biometric parameters allows detecting differences in head and eye size between both *Petaloptila* subgenera. The value for cephalic elongation (LP/HH) in the species of the subgenus *Zapetaloptila* is lower in general (0.51–0.79 for males and 0.54–0.82 for females) than the subgenus *Petaloptila* (0.64–0.83 for males and 0.67–1.14 for females), indicating that the latter has a smaller head in relation to the pronotum. The shape of the head of *P. barrancoi* (Figure 11A) is more elongated and also shows a reduction in ocular size in relation to that of *P. aliena* (Figure 11B). The head of the former is ovoid, the distance between the lower edge of the insertion of the antenna and the front-clypeus groove is greater, and the length of the eye is 1/3 of the head (greater for *P. aliena*).

Eyes are smaller and narrower in *Zapetaloptila* (Figure 11A) by presenting higher values for HH/HE (2.96–4.44 for males and 3.00–4.53 for females) than *Petaloptila* (2.30–3.37 in males and 2.50–3.37 in females) and for HE/WE (1.10–1.91 in males and 1.14–2.00 in females) versus the subgenus *Petaloptila* (1.27–1.67 in males and 1.19–1.50 in females).

**Figure 12:**
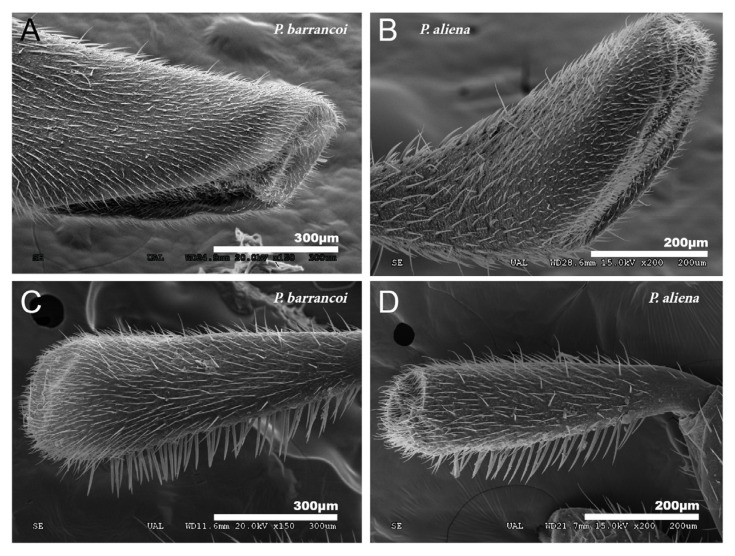
Palps. (**A**) *P. barrancoi* has a more elongated maxillary palp with a lateral sensory portion, while in *P. aliena* (**B**) the sensory portion is located more distally. In the case of the labial palps, the result is similar, (**C**,**D**), respectively.

The conformation of the mouthpart appendages, both the maxillary and labial palps, show subgeneric differences that also denote elongation. *P. barrancoi* has a more elongated maxillary palp with the sensory portion partially lateral (Figure 12A) as opposed to the more distal situation in *P. aliena* (Figure 12B). The sensory portion of the labial palp shows a similar trend (Figure 12C,D respectively).

#### 3.3.2. Depigmentation

**Figure 13:**
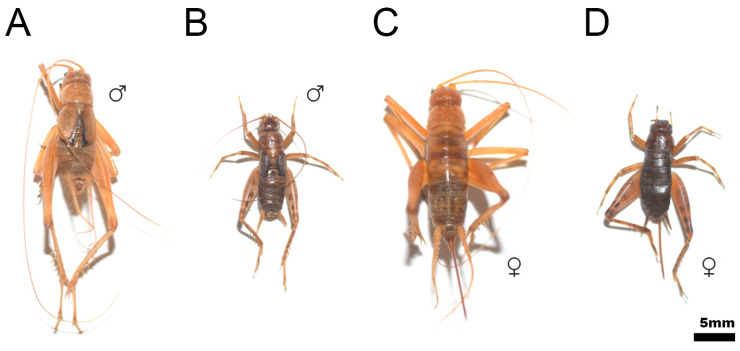
Coloration of the type species of the subgenera *Zapetaloptila* ((**A**,**C**), male and female of *P. barrancoi*) and *Petaloptila* ((**B**,**D**), male and female of *P. aliena*). Specimens were preserved in 70% alcohol of the orthoptera collection of the Entomology Laboratory of the Biology and Geology Department of the University of Almería. (**A**) *P. barrancoi*, Sima del Aire (Sierra de Gádor, Almería, Spain), 27/08/2013; (**C**) *P. barrancoi* Sima Puntal (Sierra de Gádor, Almería, Spain), 27/08/2013; (**B**,**D**) *P. aliena* Serrella (Sierra Serrella, Alicante, Spain), 10/2012. In all cases: P. Barranco coll. and det.

After numerous studies with different species of *Petaloptila* [29,30,31], and a review of the collection material, we have found that the coloration of species of subgenus *Zapetaloptila* is pale (e.g., Figure 13A,C) in general, as opposed to those of the subgenus *Petaloptila* (e.g., Figure 13B,D) which are dark or very dark. Type species of both subgenera are chosen to illustrate difference size and depigmentation. In addition, the localities chosen are from the same *terra tipica* province.

#### 3.3.3. Elongation of Appendages

Appendage elongation in both PF/LP and PT/LP ratios is higher for subgenus *Zapetaloptila* (Table 4). The range of PF/LP for subgenus *Zapetaloptila* ranges from 3.36–5.68 for males and 3.71–5.86 for females, versus 3.20–4.17 for males and 2.66–4.47 for females in subgenus *Petaloptila*. Similarly, hind tibiae are longer in subgenus *Zapetaloptila* with PT/LP values of 3.08–4.86 for males and 3.04–4.73 for females, versus the subgenus *Petaloptila* (2.16–3.10 for males and 1.84–3.14 for females). 

The Kruskal-Wallis test shows statistically significant differences in all the variables analyzed between subgenera in females, while in the case of males, there are no significant differences in the reduction in ocular size, HE/WE (*p*-value: 0.054713 > 0.05). The results are reported in Table 4.

## 4. Discussion

### 4.1. Cuticular Structures

The species included in this study are predominantly ground crickets with no flight capacity or stridulation. However, they retain tegmina, which, as mentioned throughout the study, have been modified in most of the species as a secondary sexual characteristic.

Desutter-Grandcolas [74] makes an extensive comparative study of the morphology of the tegmina of several species of crickets of different subfamilies. She describes the different types of veins and the cuticular microsculpture of both the dorsal and ventral side. Most of the species present a hexagonal microsculpture delimited by more or less patent ridges, and dorsoventral asymmetry is generalized, something similar to what our results show. However, this author does not indicate for any of the species the presence of orifices, pores, or glandular grooves as those observed in this work. This author relates the development of the hexagonal reticulum to rigidity and the development of veins to flexibility. However, this mosaic is generalized in the integument of insects [75,76,77]. It seems to be constituted by the cuticular portion of each epidermal cell that overlaps like tiles and in whose distal edge is frequent, the presence of blunt spines [75]. In the present case, we are dealing with non-canorous species (those that do not stridulate), so there are no cells, veins, or more or less flexible and vibratile zones. The function of tegmina is very different from most crickets, so the appearance of secretory structures seems to be the adaptive criterion that justifies their existence and modification. The existence of both secretory grooves and secretory pores has been detected.

It has been observed that in tegmina with a lower degree of cannibalism, bites are detected at the level of the internal apical gland openings (Figure 6A). Progressively, consumption increases up to the distal quarter of the tegmina (Figure 6B). This seems to suggest that, as in other cricket species [33,34,35], the male secretes a substance that is offered to the female, which sometimes exceeds consumption by devouring even the tegmina. As shown in Figure 6C, the female positions herself on the male’s dorsum, and the male orients his tegmina by turning them forward, exposing his ventral side, so that the apex of the tegmina is at the level of the female’s mouth. This form of mating coincides with that described by Boldyrev [33] for a species of a closely related genus, *Ovaliptila buresi* (Mařan, 1958), whose males also turn the tegmina anteriorly on the pronotum, exposing the metanotal glands to the female. These produce viscous, dark, and translucent secretion, which the female removes from the exposed ventral surface of the upwardly rotated tegmina just before and during copulation [33]. Lopes-Andrade and Sperber [34] also indicate, by means of a morphological study with SEM of the metanotal glands, the production of volatile substances in these structures in crickets of the family Phalangosidae.

Different authors have pointed out that male crickets of other subfamilies have developed different strategies for the same purpose; they secrete substances to feed the females during mating in order to prolong it and allow the transfer of the spermatophore. There are four places where the gift substances are produced [56]: in the dorsal thoracic glands, in the metatibial glands, in the glands on the outer dorsal surface of the tegmina, and on the entire outer surface of the tegmina, which form a vessel-like structure. The substances produced are consumed in situ [34], i.e., the females come to devour them in these male glands: suprathoracic [35] or metatibial [78,79]. They can also be consumed ex-situ so that the substances adhere from the dorsal glands to the inner surface of the tegmina, where they are exposed to the male during mating and are consumed there [36].

In *Petaloptila*, the existence of apical glandular openings and pores on the tegmina is demonstrated. The former are just at the level of the female’s mouth during mating (Figure 6C), and secretion (deposition of substances that have a gel-like texture when hydrated) (Figure 2A,B) and more or less partial ingestion of the tegmina (Figure 6A,B) have been observed. Consumption by the female beyond secretion could provide her with hemolymph supply as in some *Nemobiinae* [79]. We cannot rule out the possibility that this could be a key feature of male competition for access to females.

Laboratory breeding of *P. barrancoi* has shown that copulation in this species is performed as usual in crickets [51], with the female on the male’s dorsum [80]. Thus, the male turns the tegmina forward, exposing the ventral surface of the tegmina and the internal margin to the female (Figure 6C). The existence of dorsal glands on the male, which the female rubs or palpates, are common in Orthoptera, in which the female mounts the male during copulation [81,82]. Boldyrev [33] describes in detail the courtship and mating of *Ovaliptila buresi*; the male secretes a substance from the inner side of his tegmina that the female consumes. It is a species closely related to the genus *Petaloptila*. Males of the species *Nemobius sylvestris* (Bosc, 1792) secrete a substance on the dorsum of their right tegmina that is actively palpated by the female during copulation [78,83]. *Nemobius interstitialis* Barranco, Gilgado, and Ortuño, 2013 presents a total loss of stridulatory capacity, and its tegmina have a proliferation of secretory pores much higher than its congenera [84]. Lopes-Andrade and Sperber [34] have found in phalangopsis crickets, by electron microscopy techniques, the existence of a multitude of pores in the meso- and metanotum that secrete non-volatile substances.

According to Otte [85,86], cricket species that have lost the ability to hear are those that have also lost the ability to fly. There is no tympanum as it is normal in species that do not stridulate and given their way of life (Table 3). The presence of a group of campaniform sensilla is something that occurs in other crickets, such as those of the *Gryllus* genus studied by Eibl [45] and in other orders of insects [87].

Desutter-Grandcolas et al. [88] indicate that cricket species occupying the same habitat diverge in the development of cerci without being able to establish a fixed pattern, although diurnal species have shorter cerci than nocturnal species. In the present case (see the mode of life in Table 3), an increase in the length of the cerci is observed from epigean species (*P. fermini* and *P. aliena*), through interstitial species (*G. lanzarotensis*), to cave-dwelling species (species of the subgenus *Zapetaloptila* and of the genus *Acroneuroptila*).

### 4.2. Troglomorphic Adaptations

Most of the species considered are hypogean, from lavicolous MSS for *G. lanzarotensis* [71], to cavernicolous or colluvial and alluvial MSS for the subgenus *Zapetaloptila* to epigean and MSS (both alluvial and colluvial) for the subgenus *Petaloptila*, which move from sunset on leaf litter and bare ground (Table 3). Only troglomorphisms are appreciated in the species of the subgenus *Zapetaloptila*, in characters such as cephalic elongation, ocular reduction, greater length of the appendages, and depigmentation. These characters are shown in the same way in both species of *Acroneuroptila*, with very pale, coloration, reduction in eye size, and longer legs. *Zapetaloptila* and *Acroneroptila* show a very close physiognomy and biology in caves.

Most of the micropteran species considered in the study are hypogean, ranging from lavicolous MSS for *G. lanzarotensis* [71], through MSS or colluvial and alluvial for the subgenus *Zapetaloptila* to epigean and MSS (both alluvial and colluvial) for the subgenus *Petaloptila* [89], moving from dusk on leaf litter and bare ground. Features associated with cave living, such as reduced wings, eyes, and depigmentation, are found in all cave-dwelling Orthoptera [62], although the differences are smaller than those found in other types of insects. Our results show ocular reduction and depigmentation only in species of the subgenus *Zapetaloptila*. These troglomorphisms are to be expected since these are troglophilic species, in contrast to those of subgenus *Petaloptila*, which are mainly epigean (Table 3). Differences in the degree of troglomorphism are also observed in *Zapetaloptila*, in characters such as cephalic elongation, body size, and greater length of appendages. Although, according to some authors, elongation of appendages in cave-dwelling Orthoptera is not usually observed, and if it is, it is always in a much attenuated form [47]. As for limb lengths, it has also been shown that there are significant differences between subgenera, reaffirming the postulate that leg elongation occurs within closely related taxa [47,62,90]. The genus *Ovaliptila* has too many epigean species that live in the forest and some others are found in caves [27], but no hypogean character is mentioned or studied. These variations do not occur in all European species; for example, in two related species of the genus *Troglophilus* (*T. neglectus* and *T. cavicola*), no differences have been found in the degree of troglomorphism (body size, elongation of appendages, increased spinulation) or reductants (reduction in the eye) [91]. In this case, they are also phylogenetically close species, both troglophilic. One species, *T. cavicola*, penetrates more into the cave than the other penetrates and stays longer in the cave than the other, so it was expected to find some kind of differences. There are also cases in other Orthoptera families with species of the same genus that show different degrees of troglomorphism according to their way of life. An outstanding example is found in Carlsbad Cavern (Carlsbad, NM, USA). Three different species of crickets of the genus *Ceuthophilus* Scudder, 1862, coexist there, representing the three varieties of troglomorphic adaptations. The least cave-adapted species is the robust *C. carlsbadensis* Caudell, 1924 that is common in areas with bat guano. The most cave-adapted species, *C. longipes* Caudell, 1924, lives in remote areas of Carlsbad, where food is very limited. The intermediate species, *C. conicaudus*, is widely distributed in smaller caves throughout the park [50]. The results show differences in the degree of troglomorphism of the two subgenera, but it would be desirable to test these from precise phylogenies. In the case of crickets, although more and more work is being done on phylogenetic studies for different purposes [1,2,61,62,92,93], there are still few phylogenetic studies available to support these results. In addition, although their way of life has been updated, given the importance that the MSS is having in discovering in this environment, species previously linked only to caves, it is necessary to continue advancing in the knowledge about their way of life in order to have a more complete vision to face the issues discussed here.

## 5. Conclusions

The tegmina of the males of the genera *Petaloptila* and *Acroneuroptila* have lost their motor and stridulatory function and seem to confer a function in reproduction through the secretion of substances that they offer to the female and that she consumes even to the point of devouring the structure itself. However, this is not a common occurrence since most of the males of the different species studied do not have gnawed tegmina. Likewise, their ultrastructure shows a diversity of morphologies such as pores, secretory glands, or different types of setae. Precisely the absence of flight is associated with the disappearance of the tympanum in the anterior tibiae [86]. The loss of stridulation occurs by homoplastic convergence in both epigean and hypogean crickets [94]. A group of campaniform sensilla have been detected in all three pairs of legs. Species of subgenus *Zapetaloptila* exhibit troglomorphic characters, unlike those of the subgenus *Petaloptila*.

## Figures and Tables

**Table 1:** Species included in this study. The table shows the species studied. Tribe, genus, and subgenus are indicated for *Gryllomorpha* and *Petaloptila*. In addition, (+) is indicated if the species have been analyzed by scanning electron microscope (SEM) images and/or by biometric measurements (Biometry). If not, (−) is also indicated.

Taxon	SEM	Biometry
Gryllomorphini Saussure, 1877		
*Gryllomorpha* Fieber, 1853		
*Hymenoptila* Chopard, 1943		
*Gryllomorpha (Hymenoptila) lanzarotensis* Kevan versus Hsiung, 1992	+	−
Petaloptilini Baccetti, 1959		
*Acroneuroptila* Baccetti, 1959		
*Acroneuroptila puddui* Cadeddu, 1970	+	−
*Acroneuroptila sardoa* Baccetti, 1959	+	−
*Petaloptila* Pantel, 1890		
*Petaloptila* Pantel, 1890		
*Petaloptila* (*Petaloptila) aliena* (Brunner von Wattenwyl, 1882)	+	+
*Petaloptila* (*Petaloptila) fermini* Gorochov & Llorente, 2001	+	+
*Petaloptila* (*Petaloptila) isabelae* Gorochov & Llorente, 2001	−	+
*Petaloptila* (*Petaloptila) pallescens* Bolívar, 1927	−	+
*Petaloptila* (*Petaloptila) pyrenaea* Olmo-Vidal & Hernando, 2000	−	+
*Zapetaloptila* Gorochov & Llorente, 2001		
*Petaloptila* (*Zapetaloptila*) *baenai* Barranco, 2004	+	+
*Petaloptila* (*Zapetaloptila*) *barrancoi* Gorochov & Llorente, 2001	+	+
*Petaloptila* (*Zapetaloptila*) *bolivari* (Cazurro, 1888)	−	+
*Petaloptila* (*Zapetaloptila*) *malaciatana* Barranco, 2010	−	+
*Petaloptila* (*Zapetaloptila*) *mogon* Barranco, 2004	+	+
*Petaloptila* (*Zapetaloptila*) *venosa* Gorochov & Llorente, 2001	−	+

**Table 2:** Summary of the characteristics presented by each of the eight species analyzed by SEM. The presence (+) or absence (−) of certain structures is indicated.

Species	Tegmina Glands	Protibial Tympani	Group of Campaniform Sensilla	Dorsal Glands
*G. lanzarotensis*	−	−	+	−
*A. puddui*	+	−	+	−
*A. sardoa*	+	−	+	−
*P. (P.) aliena*	+	−	+	+
*P. (P.) fermini*	+	−	+	+
*P. (Z.) baenai*	+	−	+	+
*P. (Z.) barrancoi*	+	−	+	+
*P. (Z.) mogon*	+	−	+	+

**Table 3:** Lifestyle and distribution. Summary of the lifestyle and distribution [3] of the species studied. The reference is indicated if the lifestyle is included in the publications and with (*) if the data are the authors’ own.

Species	Epigean	Hypogean			Distribution
		MSS	Trogloxene	Troglophile	
*G. lanzarotensis*	[70]	[71]			Canary Islands
*A. puddui*				[72]	Sardinia
*A. sardoa*	[73]			[73]	Sardinia
*P. P. aliena*	[68]	[65]	[65]		Spain, France
*P. P. fermini*	[68]		[65]		Spain, Portugal
*P. P. isabelae*	[68]		[65]		Spain
*P. P. pallescens*	[68]				Spain
*P. P. pyrenaea*	[68]	[64,66]	*		Spain
*P. Z. baenai*	*	*		[29]	Spain
*P. Z. barrancoi*	*	*		[68]	Spain
*P. Z. bolivari*		[63]		[68]	Spain
*P. Z. malacitana*				[30]	Spain
*P. Z. mogon*				[29]	Spain
*P. Z. venosa*				[68]	Spain

**Table 4:** Degree of troglomorphism in the genus *Petaloptila.* The values of PF/LP, TP/LP, HH/HE, HE/WE, and LP/HH are mean ± S.D. PF/LP and TP/LP are ratios used to assess appendage elongation (PF, hind femur length; PL, pronotum length; PT, hind tibia length); HH/HE and HE/WE to assess ocular size reduction (HH, head height; WE, eye width; EH, eye height); LP/HH to assess cephalic elongation. The differences in the structures on *Zapetaloptila* and *Petaloptila* were determined using Kruskal-Wallis test. Mean values with different superscripts within the same column and for the same parameter indicate statistically significant differences (*p*-value ≤ 0.05).

Species	Sex	n	PF/LP	PT/LP	HH/HE	HE/WE	LP/HH	Coloration
*P. P. aliena*	♂	10	3.74 ± 0.18 ^a^	2.63 ± 0.22 ^a^	2.55 ± 0.19 ^a^	1.42 ± 0.15 ^ab^	0.79 ± 0.05 ^a^	Dark
*P. P. fermini*	♂	3	3.62 ± 0.40 ^a^	2.87 ± 0.21 ^a^	3.16 ± 0.21 ^a^	1.31 ± 0.05 ^ab^	0.73 ± 0.07 ^a^	Dark
*P. P. isabelae*	♂	3	3.53 ± 0.07 ^a^	2.82 ± 0.10 ^a^	3.17 ± 0.03 ^a^	1.33 ± 0.02 ^ab^	0.76 ± 0.01 ^a^	Dark
*P. P. pallescens*	♂	1	3.88 ^a^	2.92 ^a^	3.00 ^a^	1.29 ^ab^	0.76 ^a^	Dark
*P. P. aliena*	♀	10	3.71 ± 0.46 ^a^	2.49 ± 0.36 ^a^	2.66 ± 0.09 ^a^	1.35 ± 0.06 ^a^	0.81 ± 0.13 ^a^	Dark
*P. P. fermini*	♀	2	3.77 ± 0.07 ^a^	2.91 ± 0.31 ^a^	3.11 ± 0.23 ^a^	1.44 ± 0.09 ^a^	0.70 ± 0.01 ^a^	Dark
*P. P. isabelae*	♀	3	3.53 ± 0.23 ^a^	2.69 ± 0.11 ^a^	3.18 ± 0.03 ^a^	1.33 ± 0.08 ^a^	0.78 ± 0.03 ^a^	Dark
*P. P. pyrenaea*	♀	4	3.61 ± 0.25 ^a^	2.70 ± 0.11 ^a^	3.28 ± 0.07 ^a^	1.28 ± 0.07 ^a^	0.74 ± 0.06 ^a^	Dark
*P. Z. baenai*	♂	10	4.27 ± 0.11 ^b^	3.54 ± 0.13 ^b^	4.25 ± 0. 15 ^b^	1.54 ± 0.08 ^ab^	0.63 ± 0.01 ^b^	Clear
*P. Z. barrancoi*	♂	10	4.93 ± 0.59 ^b^	4.24 ± 0.47 ^b^	3.72 ± 0.24 ^b^	1.40 ± 0.07 ^ab^	0.64 ± 0.05 ^b^	Clear
*P. Z. bolivari*	♂	19	4.21 ± 0.18 ^b^	3.51 ± 0.23 ^b^	3.32 ± 0.16 ^b^	1.35 ± 0.10 ^ab^	0.70 ± 0.03 ^b^	Clear
*P. Z. malacitana*	♂	10	4.41 ± 0.03 ^b^	3.88 ± 0.29 ^b^	3.62 ± 0.22 ^b^	1.52 ± 0.17 ^ab^	0.69 ± 0.04 ^b^	Clear
*P. Z. mogon*	♂	10	4.41 ± 0.31 ^b^	3.65 ± 0.30 ^b^	3.68 ± 0.27 ^b^	1.46 ± 0.17 ^ab^	0.65 ± 0.07 ^b^	Clear
*P. Z. venosa*	♂	3	4.58 ± 0.68 ^b^	3.95 ± 0.61 ^b^	3.21 ± 0.35 ^b^	1.36 ± 0.02 ^ab^	0.67 ± 0.01 ^b^	Clear
*P. Z. baenai*	♀	10	4.15 ± 0.13 ^b^	3.45 ± 0.15 ^b^	4.31 ± 0.20 ^b^	1.50 ± 0.13 ^b^	0.67 ± 0.03 ^b^	Clear
*P. Z. barrancoi*	♀	10	4.61 ± 0.52 ^b^	3.88 ± 0.39 ^b^	3.65 ± 0.34 ^b^	1.53 ± 0.19 ^b^	0.69 ± 0.07 ^b^	Clear
*P. Z. bolivari*	♀	19	4.14 ± 0.17 ^b^	3.38 ± 0.20 ^b^	3.61 ± 0.30 ^b^	1.38 ± 0.17 ^b^	0.71 ± 0.05 ^b^	Clear
*P. Z. malacitana*	♀	10	4.24 ± 0.18 ^b^	3.59 ± 0.26 ^b^	3.68 ± 0.24 ^b^	1.43 ± 0.14 ^b^	0.70 ± 0.05 ^b^	Clear
*P. Z. mogon*	♀	10	4.45 ± 0.46 ^b^	3.70 ± 0.39 ^b^	3.75 ± 0.28 ^b^	1.63 ± 0.19 ^b^	0.66 ± 0.08 ^b^	Clear
*P. Z. venosa*	♀	8	4.01 ± 0.17 ^b^	3.46 ± 0.42 ^b^	3.50 ± 0.02 ^b^	1.49 ± 0.13 ^b^	0.71 ± 0.08 ^b^	Clear

## Data Availability

All microscopy data are available from the authors upon request.
